# P-196. Susceptibility of Bacterial Pneumonia to First Choice Antibiotics by Country: A Cross-Sectional Study

**DOI:** 10.1093/ofid/ofaf695.419

**Published:** 2026-01-11

**Authors:** Michelle C Davidson, Kevin Ikuta

**Affiliations:** David Geffen School of Medicine at UCLA, Los Angeles, CA; West Los Angeles VA, Los Angeles, California

## Abstract

**Background:**

Lower respiratory infections (LRI) caused over 3.8 million deaths in 2019 and were the leading cause of sepsis deaths worldwide. Treating LRIs becomes increasingly challenging with antimicrobial resistance, highlighting the importance of microbial identification and antibiotic choice. The World Health Organization (WHO) Model List of Essential Medicines lists ceftriaxone (CTX) with clarithromycin and amoxicillin (AMX) as first-line antibiotics for treating pneumonia. The aim of this study was to investigate the proportion of bacterial LRI cases susceptible to each of these antibiotics by country.

**Figure 1:**
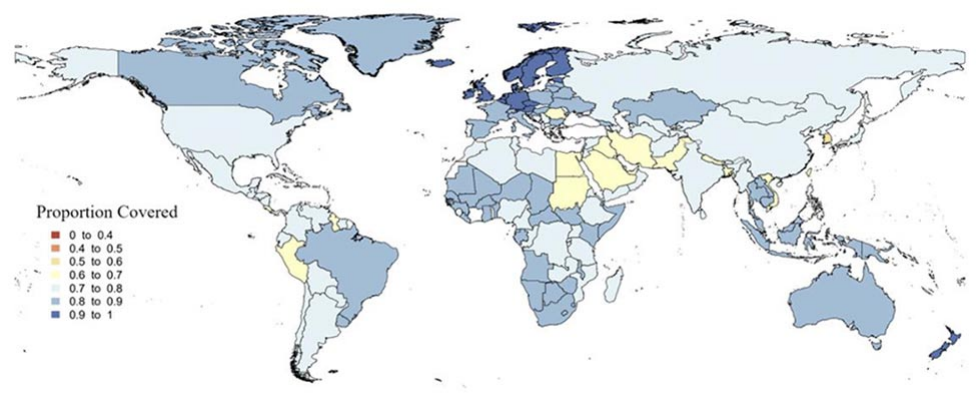
Proportion of bacteria susceptible to ceftriaxone with clarithromycin for severe bacterial pneumonia by country

**Figure 2:**
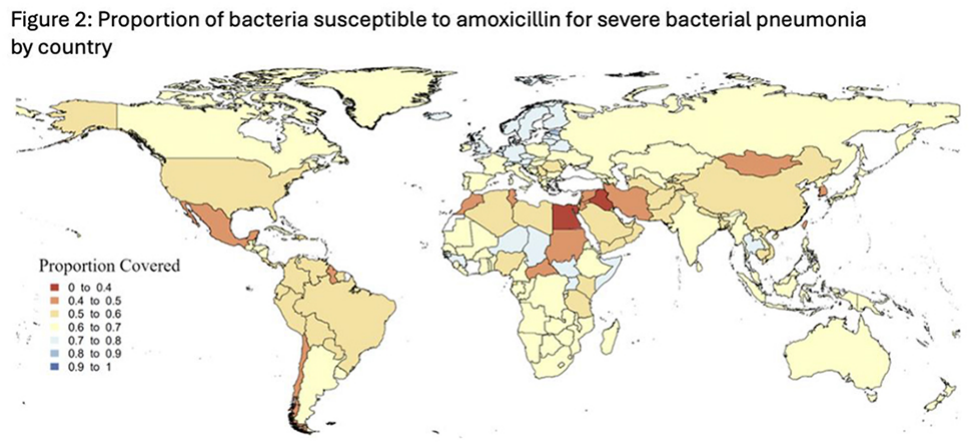
Proportion of bacteria susceptible to amoxicillin for severe bacterial pneumonia by country

**Methods:**

We used estimates from the global burden of antimicrobial resistance 2019 to determine the proportion of bacterial pneumonia cases susceptible to CTX with clarithromycin or AMX. We extracted the number of pneumonia cases by country, the country specific pathogen distribution for LRI, and the proportion of pathogens resistant to each antibiotic. We then categorized countries into seven groups based on proportion of cases susceptible to CTX with clarithromycin or AMX (0-0.4,0.4-0.5,0.5-0.6,0.6-0.7,0.7-0.8,0.8-0.9, and 0.9-1.0) and created two maps to display the categorization of each country for each antibiotic.

**Results:**

Estimates were available for 204 countries with 37.2 million global cases of bacterial pneumonia in 2019. An estimated 25.6 million cases were susceptible to CTX with clarithromycin (average susceptibility 78.9%) and an estimated 21.6 million cases were susceptible to AMX (average susceptibility 61.5%). All countries had greater than 50% susceptibility to CTX with clarithromycin, with the largest group (99 of 204) having 70-80% susceptibility. AMX had a wider range of susceptibilities, with the largest group (93 of 204) having 60-70% susceptibility.

**Conclusion:**

The ability to successfully treat bacterial pneumonia with AMX or CTX with clarithromycin varies by country. While these are recommended as first-line antibiotics for pneumonia by the WHO, these recommendations do not account for local variations in the distribution of responsible bacteria and antibiograms. Given the burden of pneumonia worldwide, providing successful antibiotic treatment is essential and could be improved by tailoring treatment based on local resistance patterns.

**Disclosures:**

All Authors: No reported disclosures

